# Time-of-Flight Neutron Diffraction (TOF-ND) Analyses of the Composition and Minting of Ancient Judaean “Biblical” Coins

**DOI:** 10.1155/2019/6164058

**Published:** 2019-03-03

**Authors:** Stephen E. Nagler, Alexandru D. Stoica, Grigoreta M. Stoica, Ke An, Harley D. Skorpenske, Orlando Rios, David B. Hendin, Nathan W. Bower

**Affiliations:** ^1^Neutron Scattering Division, Oak Ridge National Laboratory, Oak Ridge, TN 37831, USA; ^2^Materials Science and Technology Division, Oak Ridge National Laboratory, Oak Ridge, TN 37831, USA; ^3^American Numismatic Society, New York, NY 10013, USA; ^4^Chemistry and Biochemistry, Colorado College, Colorado Springs, CO 80903, USA

## Abstract

TOF-ND elastic scattering of thermal neutrons offers some important advantages over X-ray diffraction (XRD), X-ray fluorescence (XRF), and metallography for the study of archaeological and numismatic problems. Traditional analytical methods are usually destructive and often probe only the surface. Neutrons deeply penetrate samples, simultaneously giving nondestructive bulk information about the crystal structure, composition, and texture (alignment of crystallites) from which thermomechanical manufacturing processes (e.g., cast, struck, or rolled) may be inferred. An analysis of the metal composition and minting processes used for making ancient Judaean bronze and leaded bronze coins from first century BCE and CE is used as a case study. One of the first ND analyses of the temperature used for striking bronze coins is also presented.

## 1. Introduction

Neutron sources with sufficient flux intensity for practical neutron diffraction (ND) studies of small cultural objects have only become available in the last decade or two [1]. These fluxes can be achieved with nuclear reactors or accelerator particle beams that knock neutrons from nuclei in a target by a process called spallation. Pulsed neutron sources allow for very efficient and low background measurements with time-of-flight (TOF) methods that provide wavelength-resolved diffraction measurements across a broad band of wavelengths. This approach is used at the high flux, spallation neutron source (SNS) VULCAN instrument at the Oak Ridge National Laboratory (ORNL) [2]. It can simultaneously and nondestructively probe materials' crystal structures, compositions, and grain orientations, making it particularly valuable to analytical chemists and materials scientists who need to examine the entire volume of cultural objects.

Archaeological materials characterization via ND can be used to help determine the bulk composition hidden by corrosion [3, 4], for help with authentication, for reconstructing past technologies [5, 6], and for developing conservation plans by identifying artifact instabilities, such as internal corrosion. Studies using multiple techniques have included metallography, X-ray fluorescence (XRF), X-ray diffraction (XRD), and modern reference samples with known thermomechanical histories that help researchers interpret ND analyses [1, 7–9]. Despite progress for a number of artifact types, relatively few ND studies have been applied to numismatic questions. These include checking authenticity, identifying methods of minting, and determining changes in composition of silver coins from different eras and regions [10–13]. However, previous studies using ND for the most common ancient coinage alloys, copper-tin and leaded copper-tin (Pb-Cu-Sn) bronzes, appear to have been limited to a total of 20 late Roman coins [14–16]. To our knowledge, only one study has used ND analyses to infer whether ancient coins were struck while the metal was hot, and that was using silver coins [17]. Hot striking offers advantages in terms of the hammer force needed to produce an image, but it also affects the rate of coin production. The degree to which hot striking was used is an open question in numismatics.

In this study, we examine 28 bronze coins with different amounts of Pb from Judaea minted under different authorities during the first centuries BCE and CE. We use multiple techniques to interpret results from the different methods, and we use the analyses to deduce whether hot striking of bronze coins was common in this era and locale. We open our case study with an overview of the method's basic principles.

## 2. Background

### 2.1. Neutron Diffraction

There are many kinds of neutron diffraction instruments for probing materials [18], and a number of texts [19–21] and monographs [22, 23] summarize their principles. TOF-ND with instrumentation such as the VULCAN used in this study has a number of similarities to conventional X-ray powder diffraction, but also some important differences that go beyond the obvious difference in their beam sources. Both give information about the crystal structure, including the distances between the atoms in a solid that can be used to identify the elements and molecules that are present. Relative peak intensities for both are related to the relative quantities of different molecules and to the orientation of larger domains that hold them, such as crystallite phases that are preferentially aligned in one direction. The distributions of orientations are called textures. Peak widths for both XRD and ND are affected by instrumental parameters, composition, residual microstrain, and crystal grain size, with larger crystal grains giving narrower peaks.

When applied to crystalline materials both ND and XRD produce Bragg peaks. The probe can be thought of as a wave with wavelength, *λ*, that reflects from planes of atoms separated by a distance *d*. Constructive interference of waves reflected from different planes creates strong signals when the angle of reflection *θ* satisfies Bragg's law, 2*d* sin *θ* = *nλ*, where *n* is a positive integer [24]. For polycrystalline samples, the resulting spectrum is usually presented as a plot of counts versus *d*, *θ*, or 2*θ*.

Fundamentally, ND differs from conventional XRD in how neutrons and photons (or electrons) interact with matter. X-rays are sensitive to charge distributions and interact with the electron cloud around atoms [22]. Therefore, X-rays are most sensitive to elements with large atomic numbers, and XRD patterns are relatively insensitive to the distribution of atoms of elements with small differences in atomic number. Conversely, neutrons are sensitive to nuclear interactions with the atom's nuclei, characterized by a scattering length that depends in detail on the specific isotope and spin state of the nucleus [25]. The actual scattering strength is given by a scattering cross section related to the square of the scattering length. Since the cross sections vary widely for both light and heavy atoms, neutrons are sensitive to elements with both small and large atomic numbers and generally easily discriminate between atoms of elements with similar atomic numbers. It should be noted that the scattering cross sections are different from the neutron capture cross sections important to neutron activation analysis. Both are needed for calculations when determining how long a ND analysis will take and how long samples need to be held for any residual activity to decay to background levels.

**Figure 1:**
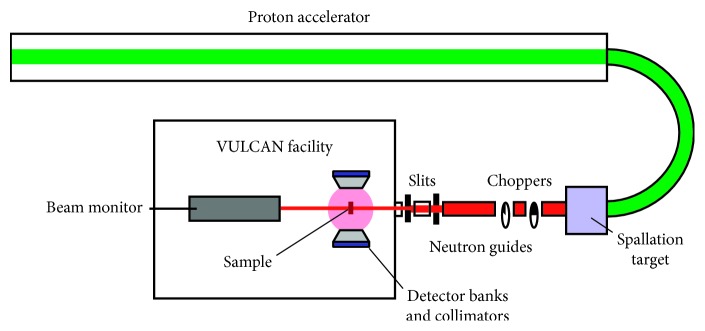
Schematic of the ORNL Spallation Neutron Source and VULCAN instrument facility. A proton beam knocks neutrons from atoms in the target, and these are pulsed at different frequencies by the choppers and guided to the sample where they are diffracted. Their time of flight determines their wavelength for Bragg scattering (see Figure 2 for details of the VULCAN diffractometer).

**Figure 2:**
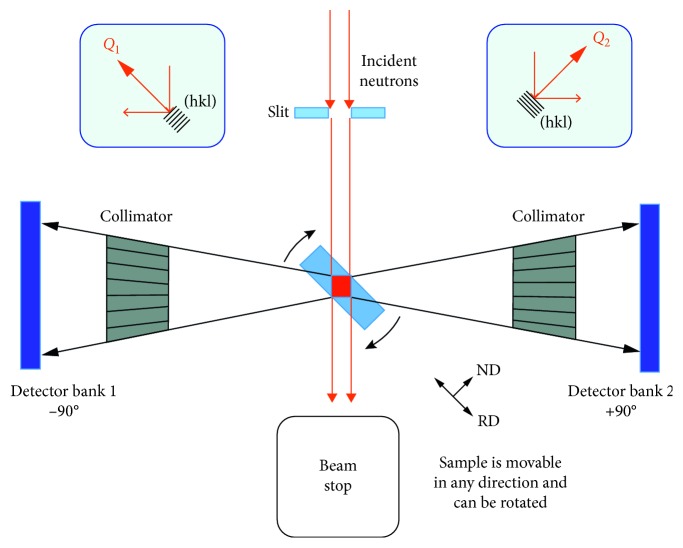
Top view of the ORNL TOF-ND VULCAN diffractometer and sample placement (not to scale). The simultaneous collection of diffraction spectra from two detector banks allows determinations of element and phase percentages using Rietveld refinement. The *X*, *Y*, *Z* movable and rotatable sample holder provides multiple angles in the *XY* plane for partial texture analyses and a computer-controlled sequential analysis of the coins (the coins are stacked vertically in the *Z*-direction).

TOF-ND makes use of the fact neutron wavelengths are inversely proportional to their velocity, *v*: *λ* = *h*/*mv* = *ht*/*ml*, where *h* = Planck's constant, *m* is the mass of the neutron, *t* = the TOF, and *l* = the neutron travel distance. Pulsed neutron sources like the SNS at ORNL produce pulses with a broad band of wavelengths. The neutrons are produced when protons from an accelerator impinge on a source target, spalling neutrons that travel from the source, diffract from the sample, and eventually reach the banks of detectors (Figure 1). Thus, the travel time, *t* = 2*mld* (sin *θ*)/*h*, depends on the wavelength, enabling a highly efficient simultaneous acquisition of diffraction patterns for many different wavelengths. A schematic diagram of the VULCAN instrument detail is shown in Figure 2.

In general, the scattering and absorption of neutrons is much weaker than that of conventional X-rays, and consequently, with the exception of a few special elements, the penetration depth of neutrons into a material is much deeper than that of X-rays, (ca. 0.1 m versus 4 *μ*m in bronze). This confers the advantage that neutron diffraction gives an excellent representation of the bulk composition of a material. With the TOF method nondestructive analyses of the bulk composition, inhomogeneities and textures are measured simultaneously, requiring fewer measurements and less time than conventional methods take to obtain comparable information by destructive means. Furthermore, though not used in this study, samples can be analyzed at the same time that they are being mechanically worked [26] and/or heated [27]. However, the relatively low scattering efficiency and count rates of neutrons can necessitate longer analysis times.

### 2.2. Crystallographic Texture

A metal object produced by cold casting from molten metal can have an initial texture that depends on its basic crystal structure (e.g., face-centered cubic, FCC) and crystal growth conditions, including the direction of any temperature gradient [28]. Mechanical working of the metal by drawing, rolling, or hammering it will force the metal's crystallites to plastically deform by sliding along crystal planes in specific directions (called Burgers vectors). Which of these directions are activated depends on the intensity and direction of stress, as well as the orientation of the grains [29]. As the sliding process is highly anisotropic, the plastic deformation will induce a steady rotation of grains. This process creates work hardening of a metal that results from the increase of dislocations and their density. Annealing obscures the deformation effects through the processes of recovery and recrystallization, and the final texture of the object will exhibit convoluted features, usually with smaller crystallites [30]. Ultimately, the combination of working and heating creates new textures that affect the relative ratios and widths of the peaks in a diffraction spectrum. These differences are used to infer the thermomechanical history of the object [31–34].

**Figure 3:**
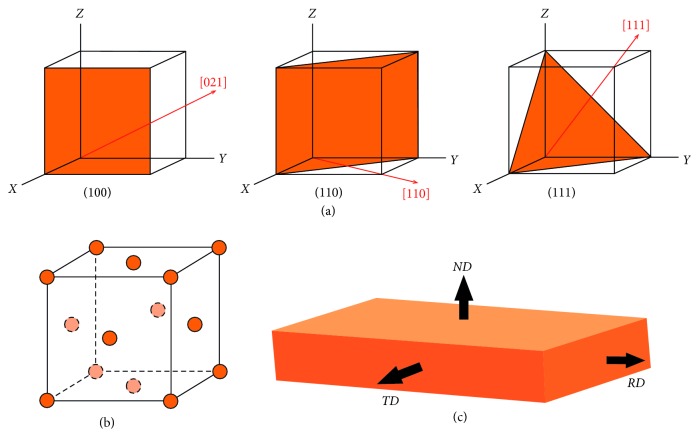
(a) Examples of Miller indices for planes (in orange) and vectors (in red) for a simple cube, (b) face-centered cubic crystal (FCC), and (c) definition of the metallurgical directions of a sample that has been worked: ND = normal direction, RD = rolling direction, and TD = transverse direction.

While a full treatment of XRD [24], XRF [35, 36], crystallography [37], metallography [38], and texture analysis [39] is beyond the scope of this paper, it will be useful to introduce some terminology and one of the more popular ways of illustrating the type and degree of texture formation found in an object: the inverse pole figure (IPF) [40]. First, the different densities and angular relationships of planes of atoms in a crystal are what scatter neutrons and X-rays so they are captured by the detector(s). The planes in a crystal unit cell (Bravais lattice) are denoted in Cartesian coordinates by their Miller indices, where h, k, and l are small integers (Figure 3(a)). Square brackets [hkl] are used to indicate the vector directions perpendicular (normal) to a unit cell's planes, and lattice planes are indicated by (hkl) (the entire set of vectors and planes with the same symmetry is denoted by <hkl> and {hkl}, respectively).

Thus, one side of a simple cube will have the plane (100), another orthogonal to it will have the plane (010), and a third orthogonal to the first two will have the plane (001). The plane (111) cuts the cell in half diagonally, while a (102) plane will span two unit cells before it intersects the corners (Figure 3(a)). While there are no restrictions on the Miller indices for primitive (simple) cubic crystals, FCC crystals (Figure 3(b)) will have reflections only from planes with Miller indices that are all even or all odd. Thus, a diffraction spectrum of an FCC crystal should not have peaks that correspond to reflections from the (100), (110), (210), or (211) planes, but it should have peaks for the (111), (311), (331), and (531) planes (the corresponding normal directions for the planes that produce peaks are [111], [113], [133], and [135], respectively).

The perpendicular to the principle surface of a rectangular object is called its “normal direction.” We will abbreviate it with an italic *ND* to differentiate it from neutron diffraction, ND. There are two other directions, each orthogonal to the others (Figure 3(c)). These are the rolling direction (*RD*) and the transverse direction (*TD*). Striking a coin from a cast blank disc will induce compression of the metal along the *ND*. Minting coins using rollers (as was done during the Medieval Ages) will create forces along both the *ND* and *RD*. While striking or rolling a metal will not change its crystal structure, the larger crystallite domains that hold the unit cells will be affected [41]. Thus, the interlocking dendritic FCC crystals of Cu that have different amounts of Sn in solid solution with Cu will be broken and bent in ways that reveal the direction of the applied forces. The resulting orientations comprise the textures that are measured.

There are two common ways used to illustrate textures found in a worked metal: pole figures and orientation distribution functions (ODFs). Pole figures are stereographic projections of the three dimensional distribution of a texture onto a two-dimensional plane. Just as projecting a map of the surface of the world onto a page distorts the shapes of the continents, projecting a 3D texture onto a plane distorts the resulting image. Because of the high symmetry found in the cubic system that includes FCC metals like Cu, Pb, and Ag, full pole figures and ODFs are often more than is needed to reveal a FCC's thermomechanical history. Sn does not have a FCC crystal structure, but up to 15.8% Sn by mass can dissolve in Cu, forming solid solutions with Cu that are FCC crystals (*α*-phases). Even higher amounts of Sn that form *β* and *γ*-phases have body-centered and simple cubic structures, respectively, while its *ε*-phase is orthorhombic [42]. Inverse pole figures (IPFs) that display only one of the 24 equivalent triangular projections that make up a full pole figure is usually sufficient for how a FCC metal was worked, especially with uniaxial deformation (e.g., compression or drawing of the metal).

**Figure 4:**
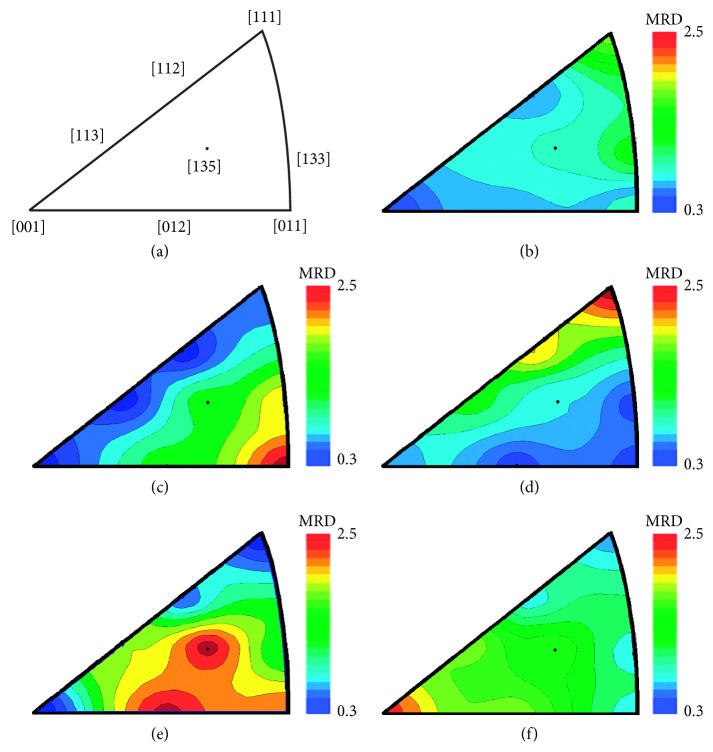
Simulated IPFs (inverse pole figures) expected for FCC (face-centered-cubic) bronze: (a) key to the fiber directions; (b) cast, thin FCC metal; (c) after compression or striking (brass or Goss texture); (d) with a *γ* fiber texture often found in extruded metal; (e) after rolling; and (f) after annealing (simple cube or *β* fiber texture). Repeated bending introduces a texture similar to rolling. Contours are in units of multiples of a random distribution.

Eight crystal vectors: [001], [011], [111], [012], [112], [113], [135], and [135] are enough to adequately construct an IPF for FCC crystals [43]. Although other vectors and projections can provide additional information, the simulated *ND* perspective in Figure 4 illustrates the different IPFs that are expected to result from casting, extruding (e.g., to make wires), hammering, rolling, and annealing ancient, small bronze coins [44]. A random “powder” distribution is taken as the reference point, and textures that develop along the different vectors are plotted in units that are “multiples of a random distribution” (mrd). The contours (or colors) in these plots reveal how strongly one direction or another is favored by the textures that develop during subsequent working and annealing of the metal. Clearly, knowledge of the context is useful for interpreting IPFs, as similar IPFs can be produced by different thermomechanical histories. Hu's treatment of texture development is useful for developing IPFs for other metal crystal structures as well [45].

Finally, although electron backscatter diffraction (EBSD) was not used in this study, for completeness, it is worth mentioning that this technique produces images of the material being examined, including the crystal orientations of the different grains in the image. Thus, the grain sizes and the microscopic texture on the *ND*, *RD*, and/or *TD* of polished surfaces can be probed using somewhat different conventions for illustrating the IPFs [46]. EBSD gives complementary surface information to that obtained from the bulk analyses obtained using TOF-ND.

### 2.3. Coin Composition and Context

Percentages of Pb vary over orders of magnitude in ancient Cu-Sn bronzes as Pb was added for a variety of reasons. These include inadvertent addition from recycling of Pb-containing metal, as a mold-releasing agent, to lower the melting point and increase the fluidity for casting [47], to make striking of images easier, and to increase the metal mass needed to manufacture small denomination, fiduciary coins to a specified total weight (*al marco*). High-Pb (≥10%) bronze was used in castings with fine details and is currently used in some bearing alloys as Pb serves to smooth and lubricate the surfaces.

The near insolubility of Pb in Cu-Sn bronzes with more than a couple percent of Pb can create an inhomogeneous mixture [48] as Pb solidifies after the bronze phases and because of its density, often forms globules that concentrate at the bottom of a casting. These inhomogeneities can affect the mechanical properties of the resulting alloy, so high levels of Pb are absent in most ancient bronze weapons. These inhomogeneities can also create wide variation in the element percentages obtained using methods that analyze surfaces (e.g., SEM, XRF, and XRD) or small volumes (e.g., laser ablation). The analytical techniques used in this study, time-of-flight neutron diffraction (TOF-ND), X-ray fluorescence (XRF), scanning electron microscopy with an energy dispersive X-ray analyzer (SEM-EDX), and light microscopy (metallography), include both bulk and surface analyses and both destructive and nondestructive approaches.

**Figure 5:**
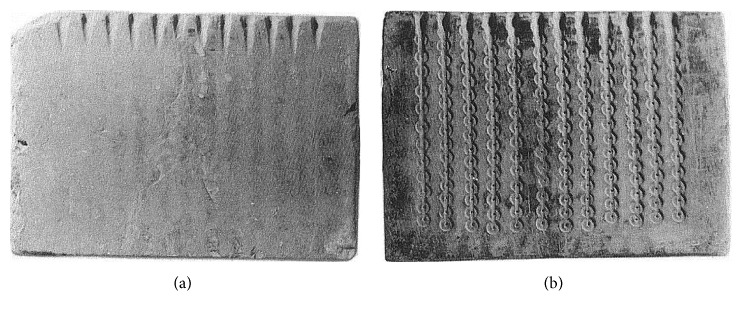
Chalkstone mold found at Khirbet Rafi', Israel, used to cast coin flans (blanks) (photo from Ancient Jewish Coins by Ya'akov Meshorer/Israel Antiquities Authority, published by Amphora Books, copyright 1982, used with permission).

Low denomination Judaean bronze coins (called prutot) were minted at various times throughout the first centuries BCE and CE when metal supply and production processes may have been influenced by different political and geographic alliances [49]. Alliances during this time alternated between the Romans and various rulers to the east and south with intermittent periods of independence. Both the numismatic and archaeological evidence [50] indicate that during this period in Judaea, the flans (blanks) from which coins were struck were made using chalkstone molds (Figure 5). In most cases, only one side of the molds had coin-size depressions cut into it using a drill. A groove was then cut between the depressions and the molds were clamped together and set on end so that molten bronze poured into it created a strip of flans with a sprue connecting them. What is not fully resolved is whether the strips were then cut apart with a chisel or blacksmith's (tinner's or cross peen) hammer followed by striking, or whether they were struck while still connected [51], though the latter seems likelier as it is an easier way to strike coins quickly [52]. There is also uncertainty about whether the flans were cold struck or whether they were struck hot, or even reheated before striking [53].

Thus, we do not yet have a detailed understanding of how the millions of coins minted throughout the Roman Empire were made. Because of the reasons noted above, ancient bronze coinage compositions contain Pb anywhere from less than 0.01% to as high as 20% or more. Using destructive analyses (e.g., atomic absorption), Lönnqvist suggested that at least four distinct compositions may be found [54] in the Judaean coins produced under the Roman governors. Rather than the large centralized mints that we have today, it is likely that small workshops in different locations that used different metal sources were responsible for producing the small bronze Judaean coins in this study [50, 55, 56]. While the images that were struck and the metal sources used changed when the Roman governors took over; we do not know how the Roman occupation that began in 6 CE affected the methods of production, though some differences in die axes and other parameters have been found [55, 56].

Unlike many ancient metal objects such as swords, axes, and pins that were worked with repeated hammer blows, often in cycles with annealing, coins were likely struck without subsequent annealing. The purpose of this numismatic case study was to conduct an analysis of a range of ancient leaded bronze coins that would illustrate some of the advantages and disadvantages of various traditional analytical methods that may be used to determine chemical compositions and thermomechanical histories in relation to what TOF-ND offers. The application of these methods to ancient bronze Judaean coins was also undertaken to gain insights into how coin manufacturing processes and compositions varied with shifting political boundaries and administrations during this pivotal location and period of history. Despite the necessarily limited number of samples, some broadly applicable information can be obtained.

## 3. Materials and Methods

### 3.1. Overview of the Experimental Process

Because ND currently requires a nuclear reactor or particle accelerator to obtain a sufficient neutron flux, access can be problematic. Most experimentalists need to write a proposal for beam time at one of the user facilities at various national laboratories such as at ORNL. Typically, proposals submitted to these facilities are reviewed twice a year by an external panel of scientists to determine the feasibility, safety, and merit of the proposal. If the proposal is successful, there is no cost for the beam time. An example of the steps involved in preparing proposals in the USA and help with the preliminary calculations are available online at http://www.nist.gov/ncnr/planning-your-experiment and http://www.neutrons.ornl.gov/users/proposal-types as well as at other facilities.

### 3.2. Samples

**Figure 6:**
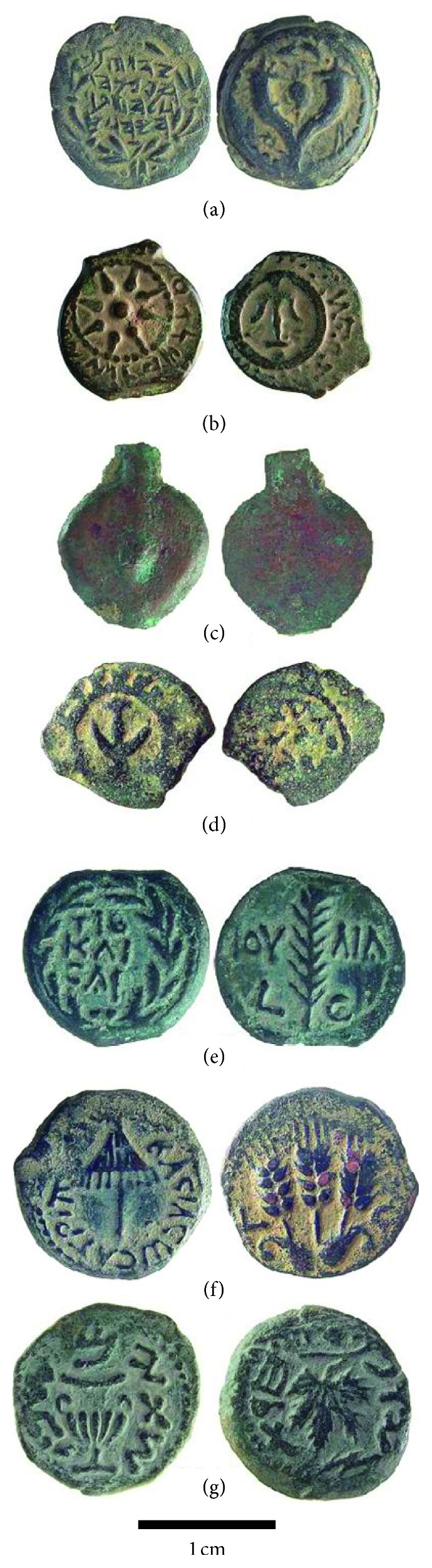
Judaean prutah analyzed by TOF-ND: (a) GBC-1133, JH = John Hyrcanus I (135–104 BCE); (b) GBC-1152, AJ = Alexander Jannaeus (104–76 BCE); (c) US = unstruck blank or flan from the time of Jannaeus and/or Herod I; (d) GBC-1174 to 1177, HG = Herod I, the Great (40–4 BCE); (e) GBC-1339, VG = Valerius Gratus (15–26 CE); (f) GBC-1244, Herod Agrippa I (37–44 CE); and (g) GBC-1360, JW = Jewish War/Revolt, year 1 (66–70 CE). GBC = Ref. [49].

All coins (exemplars are shown in Figure 6) were exported from Israel with approval of the Israel Antiquities Authority and all coins analyzed were from the authors' personal collections and were readily identifiable. The coins were physically cleaned by scrubbing with dilute dish detergent using a nylon toothbrush, rinsing with deionized water, and air-drying before analysis. Twenty-eight coins (*n* = 5 for each type in Figure 6 except for Herod Agrippa I, for which *n* = 2 and John Hyrcanus, with *n* = 1) were used for this study.

### 3.3. Microscopy and Metallography

A coin or flan of six coin types were polished on a surface, an edge, or across a connecting sprue with progressively finer SiC sandpapers (Matador Softflex 991A), ending with <5 *μ*m ferric oxide jeweler's rouge (ASTM-3E-11). The coins were examined for their microstructure and the homogeneity of Pb inclusions and internal corrosion products using a scanning electron microscope in backscatter electron mode and with an energy dispersive detector SEM-EDX (Jeol 6390LV/Oxford-INCA) for estimates of the elemental composition necessary for the proposal calculations. The polished coins were also used to obtain the crystallite size estimates. Etching was done with ammonia-peroxide for a metallographic analysis using a Unitron Mec3 [38].

### 3.4. X-ray Fluorescence and Diffraction

For the purposes of comparison to the ND data, analyses of the coins were done using XRF and XRD. XRF was conducted using a PANalytical Epsilon-5 instrument with a 100 kV Gd side-window X-ray tube with polarizing optics and five secondary targets (Al, Ti, Ge, Mo, and Al_2_O_3_) that typically provide a limit of detection of 0.1% or lower for Cu, Sn, and Pb. The current was automatically adjusted to maintain 600 W and a 50% deadtime so that there was 100 s of livetime per target. The instrument's semiquantitative mode, which is based on a fundamental parameters algorithm, was calibrated with bronzes of known composition placed in holders with polypropylene (PP) windows that were also used for the coins.

The XRD analyses were conducted using the coins that were polished for the metallographic analysis. A PANalytical X'Pert Pro XRD with an Empyrean Cu X-ray tube with a 5 mm mask and a 0.5° collimator was used to obtain XRD spectra over a 2*θ* range from 20° to 90° (0.10 to 0.44 nm) at 0.05°/s.

### 3.5. Time-of-Flight Neutron Diffraction

TOF-ND was conducted using the neutron diffractometer (VULCAN) at the spallation neutron source (SNS) at ORNL [2]. These types of diffractometers use the TOF of neutrons between the neutron source and the detector to determine precisely the velocity of individual neutrons. To this end, pulsed neutron sources (such as the SNS) are used to set the time of neutron generation, and accurate pulse detection timing allows tracking the neutron energy by recording each neutron event within a sharp time interval of 100 ns. Neutron velocity is inversely proportional to neutron wavelength, which makes its wavelength proportional to the TOF. This provides an easy way to calibrate the instrument, as position sensitive detectors are used that determine the scattering angle. In a powder diffractometer, a unique diffraction pattern (intensity versus d-spacing) is obtained from each detector bank. The sampling volume inside the specimen is defined by the cross section of the incident beam and the viewing angle of the collimators located in front of the detector banks. Specifically, the VULCAN diffractometric arrangement includes two scintillation detector banks at ±90° that allow simultaneous measurement of normal (*Q*_2_) and transverse (*Q*_1_) scattered neutrons (Figure 2) when a disc-shaped sample (a coin) is positioned at 45° in relation to the incident beam direction. The 5 × 5 mm^2^ beam that was used for our coin investigation gives a gauge volume of about 60 mm^3^ based on a typical coin thickness of 1.8 mm and a collimator field of view of 5 mm.

The sample platform with a Teflon sample holder held and automatically manipulated all 28 coins, allowing the analyses to be conducted sequentially (ca. 2 hours total per coin for the phase, composition, and texture analyses) without operator input. Low hydrogen Teflon was chosen for the holder to minimize potential background scattering of the neutrons.

Pure element powders of Cu and a mixture of Cu and Sn were compressed and analyzed to provide a random distribution of metal crystals for the mrd reference (Figure 4) for the texture analyses and to calibrate the elemental composition of the solid solution bronze phase. Rietveld refinement [57] of the diffraction spectra was conducted using GSAS software [58] to identify the crystal phases (e.g., *α*_1_ and *α*_2_ bronze). The elemental compositions (Sn versus Cu) of *α*-bronze phase was estimated from the change of lattice constant using a calibration curve (Vegard law) such as is found in [59] and confirmed by our measurements on reference samples. Selected coins were also analyzed over the 0.5–3.5 Å interval of d-spacings to access crystal planes with higher d-spacing characteristic of minor phases found in some coins.

In order to analyze the preferential orientation of grains (the crystallographic texture), coins were rotated about the vertical axis and separate diffraction patterns were recorded at 5° increments between 45° and 90° (normal to the coin face and the incident beam direction). As the coins were rotated only around one axis, complete pole figures could not be produced. However, only deep rolling is known to induce highly asymmetric texture components. In our case, *ND* is considered the axis of symmetry for the texture analysis. This approximation allows a reconstruction of *ND*–IPFs in which the texture is calculated and expressed as multiples of a random distribution (mrd) of crystals [43].

### 3.6. Statistical Analysis

Plots of the data were conducted using Excel (Microsoft Windows 10 Pro, 2016, Seattle, WA 98105, USA) and a statistical software package (Minitab ver. 18.1, Minitab Inc., State College, PA, 16801, USA). These were used to conduct statistical tests of significance (with probabilities, *P* ≤ 0.05 considered to be significant) using Student's *t*-test, Fisher's *F*-test, the chi-square (*χ*^2^) test, and Pearson's correlation coefficient, *r*, with *n* equal to the number of samples.

## 4. Results and Discussion

### 4.1. Microscopy (Metallography)

**Figure 7:**
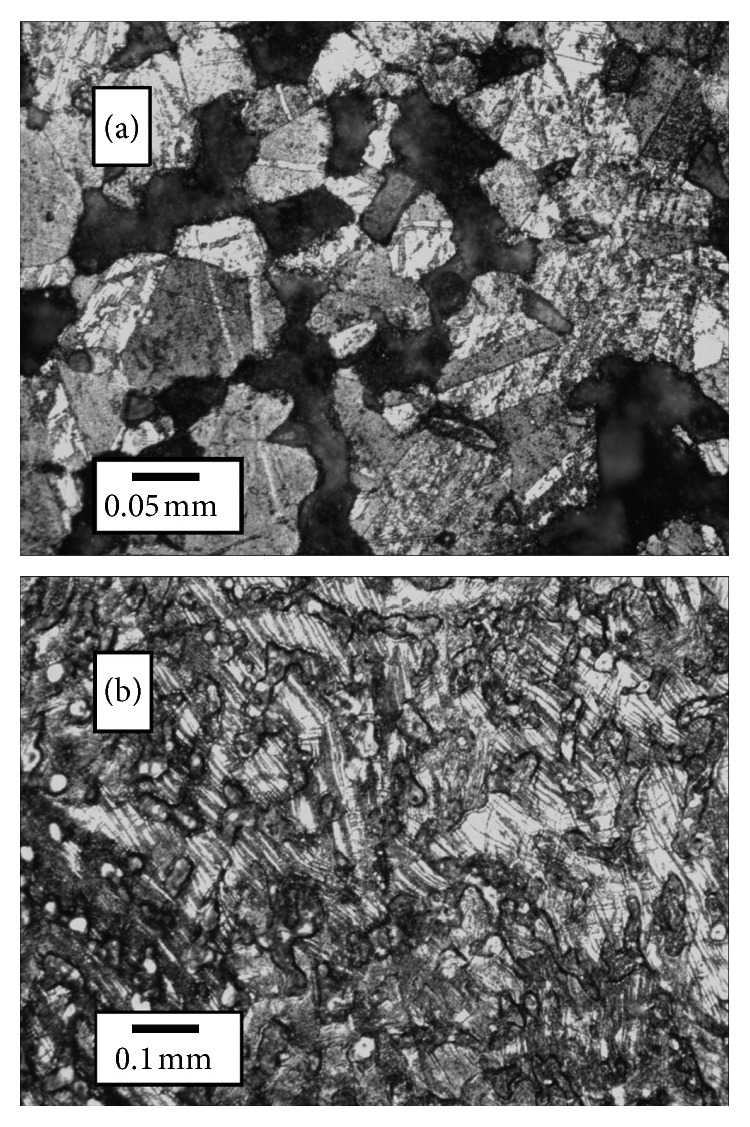
(a) Metallographic images for JW-4, with dark lead containing regions and small, rounded crystals of bronze with banding from annealing, and (b) for AJ-3 with crystal fractures and microstrain as well as bending from a cold strike below the annealing temperature similar to that found in VG-3 and VG-4.

Metallography has long been used to detect thermomechanical treatments, such as hammering and annealing [38]. Annealing can be detected with a microscope as it forms twin crystals from partial melting during a hot strike or from subsequent heating, creating parallel banding inside crystallites. This is seen in JW-4 (Figure 7(a)). Residual mechanical stresses can also be seen, such as in the bending of crystallites and the formation of dislocation fracture planes in the crystals of AJ-3 (Figure 7(b)). While these time-honored methods have provided many insights into ancient metallurgical processes, cutting and polishing even small portions of the sample are damaging and nondestructive methods are needed to study irreplaceable cultural resources.

### 4.2. Composition Analysis

Results of the ND-TOF phase analysis for the 28 flans and coins are summarized in Table 1. The percentages of the different phases estimated from GSAS refinement are somewhat imprecise due to the poor statistics of diffraction peaks belonging to minor phases. For example, less than 1% in the Pb-phase is usually set equal to zero. On the other hand, the percentage of Sn in *α*-bronze is estimated from the lattice constant [59] and the error is much smaller (<0.1%), provided the metal is a pure binary solid solution and other impurities such as Pb do not play a significant role in the average atomic structure. Although a *δ* eutectoid has been detected by ND in a few Bronze Age artifacts with Sn levels above 14% by mass [60], only Cu-Sn *α*-phases were found in the coins in this study.

**Figure 8:**
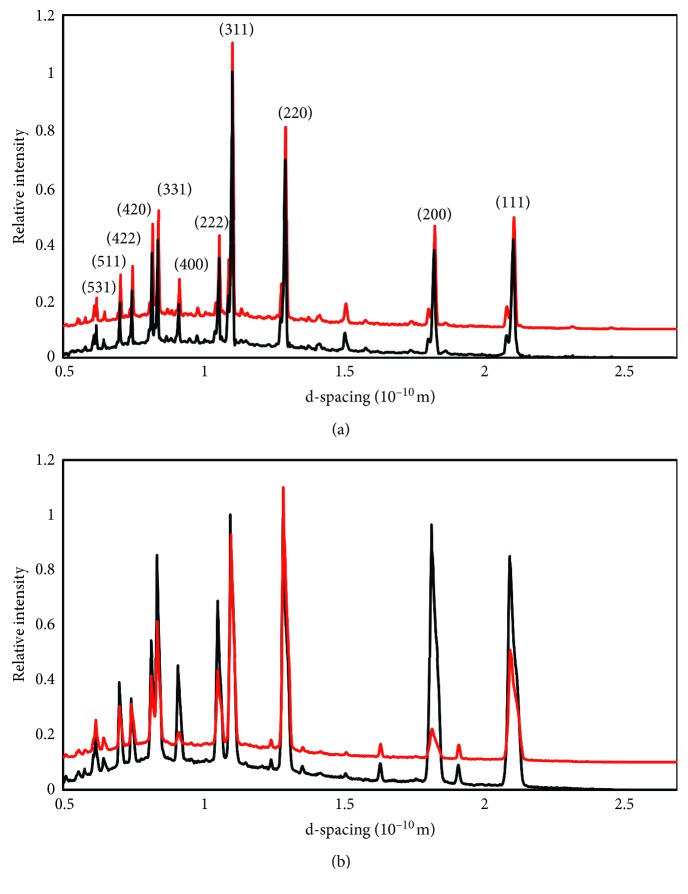
ND results from *Q*_1_ (black) and *Q*_2_ (red) for (a) JW-4, illustrating a spectrum from an annealed sample, and for (b) VG-4, a spectrum with large texture differences and significant line broadening due to factors such as phase homogeneity. *Q*_2_ is offset by 0.1 for visual clarity.

**Figure 9:**
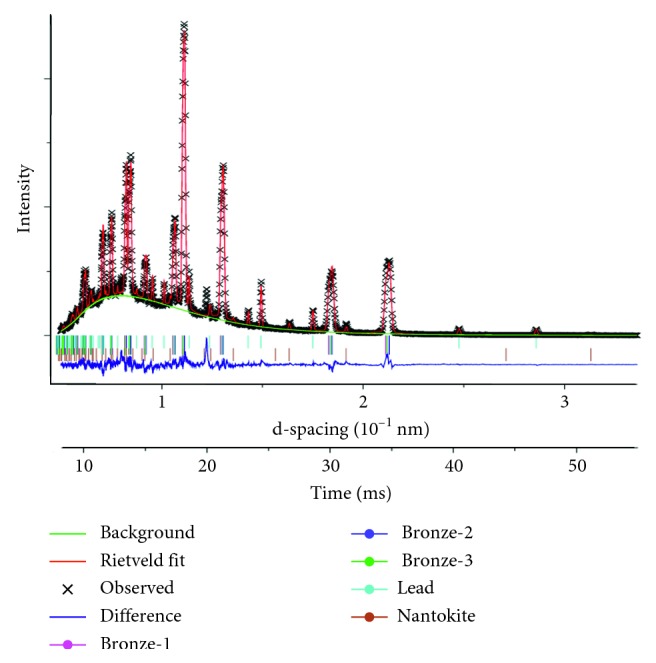
Rietveld refinement of the TOF-ND data for HG-2 with a slightly worse fit (*χ*^2^ = 5.1) than the average (*χ*^2^ = 4.6) for the coin spectra.

Three of the 28 coins exhibit significant levels (>2%) of at least one copper corrosion mineral (CuCl, Cu_2_O, or CuO). Only three or four coins have just one *α*-phase, and seven have Pb below 1%. Examples of diffraction patterns recorded by the neutron detector banks are given in Figure 8. With Rietveld refinement of the diffraction spectra (Figure 9) and using the linear relationship of the lattice parameters (d-spacing) to the composition [59], percentages of Cu, Sn, and Pb as well as the percentage of the bronze phases and corrosion or residual ore compounds were calculated.

Results of the XRF and ND-TOF elemental composition for the 28 flans and coins are summarized in Table 2. The penetration of X-rays is energy dependent, and surface roughness will affect their absorption and fluorescence emission. Thus, surface-enriched elements (Pb and Sn) are over represented in the XRF data. Even with flat, polished surfaces, Pb from the XRF analyses shows higher percentages in Table 2 than are present in the bulk of the coins obtained from the TOF-ND analyses, as Pb is preferentially forced to the surface during striking, and it is easily smeared on surfaces during polishing if care is not taken. On the other hand, bronze phases with a higher Sn to Cu ratio are harder and more resistant to abrasion, so natural wear or polishing of the surface can cause Sn levels to be higher than in the bulk composition. These results are in keeping with those of Canovaro et al. [15] for 4^th^ century Roman bronze coins, where the 22% Pb found using ND was only about half what was obtained using XRF. Despite differences (paired *t*-test, *P* ≤ 0.034, *n* = 26) in the element percentages obtained by the two methods, the XRF and TOF-ND results are significantly correlated even for the inhomogeneous element, Pb (*r*^2^ ≥ 0.36, *P* ≤ 0.001, *n* = 27).

A discriminant analysis with cross validation of the ND and XRF data for Sn and Pb in Table 2 correctly groups 85% and 90%, respectively, of the 20 struck coins for which we have five examples. Combining the bulk and surface analyses correctly grouped 95% of the 20 coins, and including the Cu data in a cluster analysis correctly clustered 100% of the coins. This implies there is useful information in the surface as well as the bulk composition. The unstruck flans do not form a separate cluster. They are closest to the Herod the Great (HG) and the Jewish War (JW) coins in their Cu-Sn-Pb composition. Because they were never struck, the images cannot tell us if they are from different rulers or times. However, previous analyses of the isotopic composition of similar unstruck flans [56] as well as the flans' masses suggest most were probably cast circa 60 BCE.

The VG coins' (dated 17/18–24/25 CE) almost complete lack of Pb is interesting. Lönnqvist has suggested the VG coins were manufactured under Pontius Pilate instead of Valerius Gratus, as an aqueduct that would have needed Pb was constructed during Pilate's administration [61]. However, we think it more likely that under Emperor Tiberius (14–37 CE), the coinage reform that began under Augustus was promulgated in some of the provinces that were gateways to trade with the East. It was at this time (18–19 CE) that Tiberius' heir apparent, Germanicus, was touring in neighboring parts of the Eastern Mediterranean. There is archaeological evidence [62] for a decree he gave in Palmyra (current day Syria) stating taxes were to be paid in Roman asses (which were a lead-free bronze at that time in Roman Syria) rather than in the local (leaded bronze) coinage. Although there is scatter in the data, other coins issued in the Levant outside of Judaea around this same time also show a corresponding dip in their levels of Pb.

### 4.3. Peak and Phase Width Broadening

**Figure 10:**
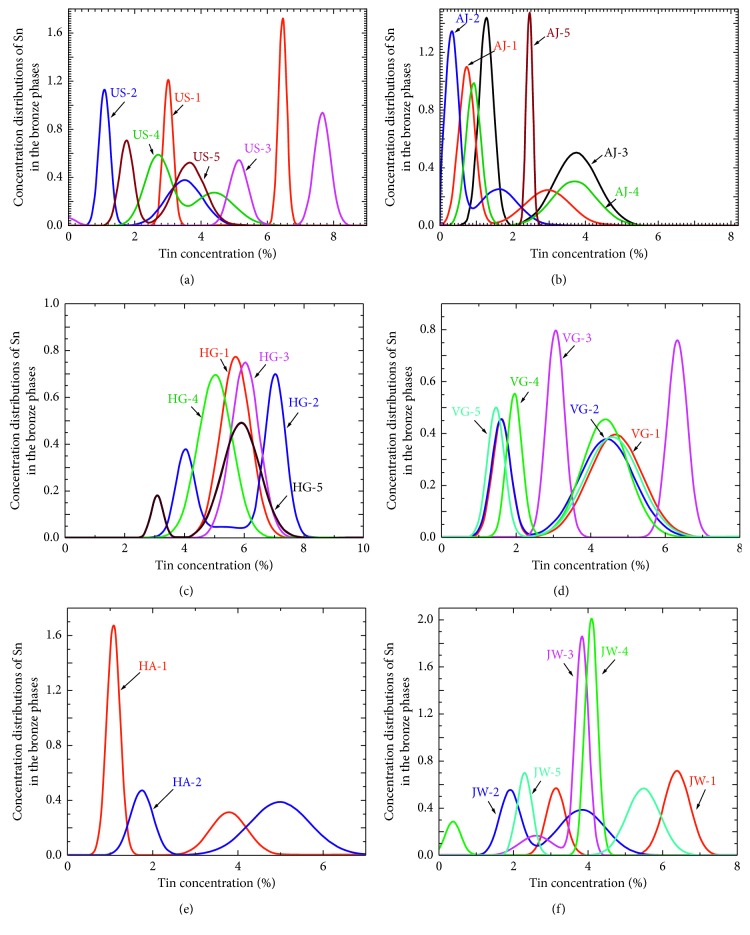
Distributions of Sn in the *α*-phases in six of the coins and flan types: (a) unstruck flans, no dates, (b) Alexander Jannaeus, 80/79 BCE, (c) Herod the Great ca. 27–22 BCE, (d) Valerius Gratus, 18/19 CE, (e) Herod Agrippa I 41/42 CE, and (f) Jewish War/Revolt 67/68 CE. The single JH-3 had a distribution very similar to HA-1, shifted slightly to a lower Sn concentration.

The peak shape of a neutron diffraction spectrum (Figures 8 and 9) includes a combination of contributions from the instrument, from the sizes of ordered domains inside crystalline grains, from residual inhomogeneous elastic microstrain in the crystallites, and from solution inhomogeneity during solidification. The VULCAN instrument resolution profile was measured with a standard Si powder and its contribution was taken into account with the GSAS refinement. Sample broadening was modeled as a Gaussian contribution for each phase separately. The coherent domain size contribution to broadening is usually considered negligible in “as cast” alloys or if only mild mechanical processing (working) has been done. Metallography, SEM, and XRD analyses confirm that the dendrites and crystallite sizes in these coins are large enough (typically ≥ 50 *μ*m) that the crystallite sizes are insignificant contributors to line broadening (the widths of the lines are inversely related to the size of crystallites for both ND and XRD [63]). Thus, crystallite size-specific broadening effects can be neglected. However, both elastic microstrain and concentration inhomogeneities affect the d-spacing, and they cannot be easily separated. Therefore, the phase widths shown in Figure 10 are in terms of % Sn, but they include all contributions except the instrument, as that was removed during the GSAS refinement.

In Figure 10, the two different *α*-phases are caused by “coring” during the formation of dendrites as the lower % Sn *α*-phase solidifies first. During cooling and solidification, more than one *α*-phase can form if the melt does not have time to come to complete equilibrium and dendrite growth promotes phase separation. Two distinct Cu-Sn *α*-phases are found in 93% of the Judaean coins in this study. The range of % Sn in the two *α*-phases fits with rapid cooling similar to chill-castings that have not been annealed (see the phase diagrams for archaeological bronzes in appendix G of Scott's text [38]). As shown in Figure 10(a), if the Sn concentration has distinct values and the broadening effect is small, the *α*-phase peaks are well separated. However, most of the samples (see Figure 10(b)) show more convoluted profiles and Rietveld refinement is necessary to retrieve the distribution of Sn concentrations.

A multiple linear regression of the phase widths versus the % Pb, % Sn, and the temperature of solidification for the struck coins indicates increasing Pb broadens the low Sn, *α*_1_-phase widths (*r*^2^ = 0.65, *n* = 22, *P* ≤ 0.001 for Pb; *P*=0.10 for Sn, and *P*=0.10 for temperature). On the other hand, the *α*_2_-phase width does not have a significant correlation with any of these variables in the struck coins (*r*^2^ = 0.22, *n* = 20, *P*=0.12 for Pb; *P*=0.37 for Sn, and *P*=0.37 for temperature). Because JW-4 is an outlier, it was not included in the regression (see below; metallography revealed that it was annealed).

Generally, a higher concentration of Sn increases the hardness of the bronze while more Pb decreases it. A harder alloy may be expected to develop more microstrain when an image is struck, assuming the alloys are at the same temperature and have an equal degree of reduction in thickness during striking. Because broadening of the *α*-phase widths are due to both residual microstrain and solution inhomogeneity during solidification, it is difficult to determine just how much of the line broadening is contributed by each.

Despite this difficulty, by comparing the Pb dependence of the line widths of the struck coins to that of the unstruck flans, we can explore whether there is a measurable additional broadening due to striking. From a multiple regression of the *α*_1_-phase widths versus the % Pb (a continuous variable) and struck versus unstruck flans (a categorical variable) in Table 1, we find there is significantly greater phase broadening for the struck coins than for the unstruck flans (*r*^2^ = 0.47, *n* = 27, *P*=0.02). An increase in the *α*_1_-phase width is expected if there is a residual microstrain component from striking the coins in addition to the contribution from solution inhomogeneity.

The Jewish War (JW) coins in Figure 10(f) exhibit a variety of phase distributions and widths. This suggests a nonuniform minting process. This may be due to limits the war with Rome had on metal supply, as the larger concentration of trace elements [64] in the Jewish War coins suggests recycled metal was being used. Furthermore, the narrow phase widths suggest JW-4 was heated subsequent to being struck and the metallography (Figure 7(a)) confirms this. Furthermore, the presence of tenorite (CuO) is indicative of heating after casting (exposure to subsequent fire and oxygen), as the Cu(II) oxidation state will equilibrate to Cu(I) when it is in direct contact with Cu metal.

The HG coins are the least homogeneous. Many of the HG coins exhibit a pure Cu-phase mixed with the *α*-bronze phases. In those instances, the ores (or metals) were probably not heated all the way to the melting point of Cu (1085°C) and sintering of the Cu by the lower melting Sn and Pb took place instead of complete melting. Similarly, the unstruck (US) coin flans exhibit a less homogeneous composition of elements and of phases. This supports metrological and isotopic analyses [56] that group the unstruck flans with late AJ or early HG coins.

Though there is a wide range in the phase widths, if we do not include the annealed coins (e.g., JW-4 and possibly AJ-5) in the comparison, the average *α*_2_-phase widths (but not the *α*_1_-phase widths) are narrower in the unstruck (US) flans than in the struck coins. This is expected if striking adds a microstrain component to the line widths. This is because the effect Pb has on line width broadening in the unstruck flans is expected to come only from Pb's effect on the cooling of the melt and the inherent disorder it creates in the formation of the *α*-phases.

The Roman (VG) coins have some of the largest phase widths. Because these coins lack Pb, the large widths may be a result of solution inhomogeneity due to rapid cooling below the relatively high annealing temperature. Alternatively, they may have been struck below their annealing temperature (a “cold strike”) and significant microstrain was created.

The phase width analysis tells us the coins' images were created by some form of mechanical working (e.g., striking or rolling) and not simply by casting. However, a texture analysis tells this story better, including which of these processes was employed.

### 4.4. Texture and Metallurgical Analyses

Unlike some of the late Roman coins examined in previous ND studies [14–16], the flans used for the Judaean prutot were cast in vertical, stacked stone molds with channels cut between them (Figure 5). The flans did not require any further flattening or annealing before being struck. This simplifies interpretations of the coins' thermomechanical histories. The sprues that can be seen and the cut marks on each side of the coins in Figure 6 indicate that all of the Judaean flans and coins in this study were produced using vertical molds.

The diffraction spectra (Figure 8) simultaneously collected from the normal (*Q*_2_) and transverse (*Q*_1_) detector banks (Figure 2) with a minimum of two beam-to-sample angles were used to calculate the texture. As noted earlier, texture is the nonrandom arrangement of the crystal grains in the alloy that arises from its thermomechanical history.

Cast FCC metals of finite size typically have a small amount of texture even before being worked [28]. If the rate of cooling is slow during casting in a mold and the heat loss is not isotropic, dendritic crystallites can grow to a significant size in a preferred direction. This may be due to a temperature gradient in the mold, as the bottom of the mold should be cooler than the top. Segregation of dense, insoluble Pb during cooling may also take place. These effects should create domains that are aligned with the casting sprues in the vertical direction in the mold. If the inhomogeneity is large enough, it will appear to be a rolling texture when IPFs are constructed from the *ND* perspective. Thus, some texture above and below 1 mrd might be expected even for the unstruck flans in Table 1.

The texture coefficients found in this study (Table 1) are small compared to the 10 mrd found in modern coins that are machine-struck using planchets cut from rolled and annealed metal strips. A third of the samples (6 of 22 struck coins and 3 of 5 unstruck flans) did not exhibit a measurable texture. The others have textures comparable to the mrds reported for five late Roman leaded bronze nummi [15], where maximum textures varied between 1.05 and 1.47 mrd. They are also comparable to the textures in modern Cu-Sn bronzes used to recreate striking processes (maxima from 1 to 2 mrd) and to the textures measured in 16^th^ century silver thalers minted by rolling and hammering [11]. The relief in the Judaean coins is comparable to modern, machine-struck coins. Hand strikes cannot achieve the same force as machine strikes, so we suggest the ancient flans were softened by the presence of Pb and/or by “hot striking.”

Salvemini et al. [17] recently used the maximum minus the minimum in the texture coefficients (a Δ texture) from a variety of ancient, struck silver coins to infer which coins were struck while hot. Although the different coin shapes required correction for absorption differences, they argued that coins with little or no texture were struck hot (ca. 700°C or above), and those with the largest texture differences were struck while “cold” (ca. 200°C for the silver coins). For the leaded bronze coins in this study, the similarity of coin morphology minimizes neutron absorption differences, but the correlation of % Sn and Pb (*r*^2^ = 0.67, *n* = 28, *P* ≤ 0.001) tends to confound striking temperature analyses, as more Sn increases the hardness of the alloy, while more Pb decreases it. Furthermore, increasing Sn lowers the temperature of solidification and annealing. The combined effects suggest minters may have controlled alloy conditions to obtain a constant hardness and thus a constant hammer force used to strike coins.

**Figure 11:**
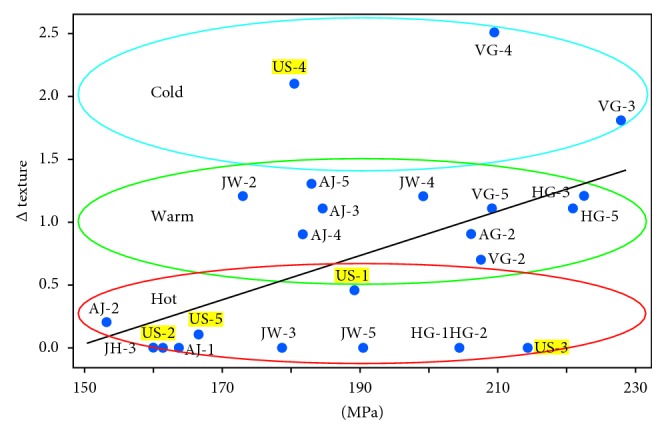
Dependence of the Δ texture (maximum-minimum in the texture coefficient) on the estimated prestrike hardness of each alloy is indicated by the regression line (*r*^2^ = 0.29, *n* = 20, *P*=0.014; unstruck flans not included). Regions of hot, warm, and cold strikes based solely on the Δ texture are indicated with ovals that are meant to focus attention and do not indicate statistical entities.

Using linear calibrations of published values for cast copper alloys [65], the bulk % Sn and % Pb in the coins can be converted to a predicted hardness in their cast metal flans before they were struck. In Figure 11, the Δ texture coefficients of the struck coins are plotted versus their predicted hardness. Most of the variation appears to be due to the hardness of the alloy, but some struck coins (HG-1, HG-2, JW-3, and JW-5) fall far enough below the line to suggest they were hot strikes. As expected, the unstruck flans do not exhibit a significant texture with one notable exception: US-4. A closer look at the source of this texture reveals that it is primarily from the Pb-phase, not the bronze phases, possibly from inhomogeneity created during pouring. It is also quite thin (<1 mm), and the [111] orientation is expected in such cases [28]. This orientation is especially favored if the primary temperature gradient is towards the flat surfaces. This would be especially true in casting of flans on a flat surface. This underscores that it is quite likely a variety of casting and minting techniques were used by ancient artisans, and analyses of multiple examples are necessary to see if these varied with time or location.

**Figure 12:**
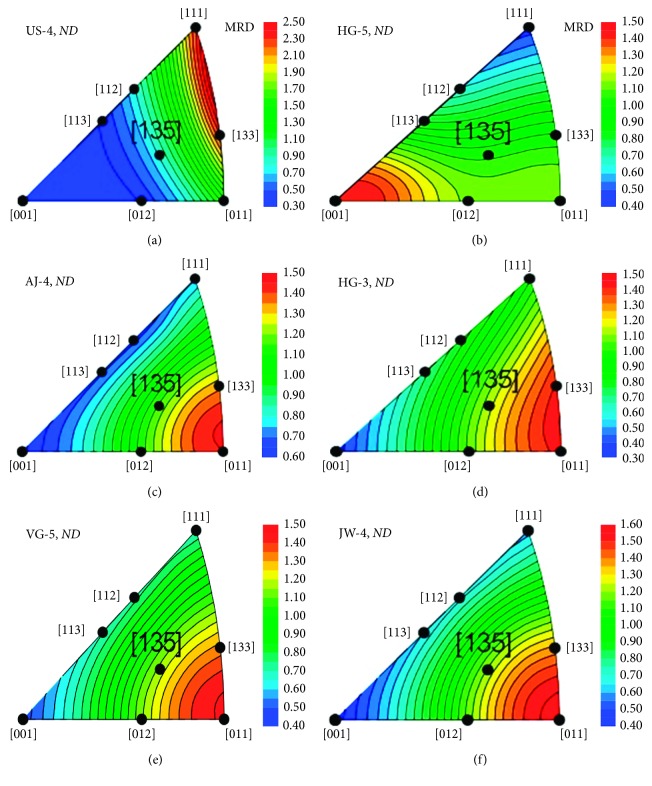
From top left: inverse pole figures for an unstruck, cast flan; a struck coin of Herod the Great (HG-5), whose figure is characteristic of a recrystallization (annealing); four coins exhibiting the expected compression figures (AJ-4, HG-5, VG-5, and JW-4) from striking. Although JW-4 was annealed or struck while hot, the compression figure suggests it was only partially annealed or was struck again subsequently. Contours are in units of multiples of a random distribution.

The inverse pole figures (IFPs) in Figure 12 tell which crystallographic directions are oriented parallel to the sample coin's normal direction (i.e., perpendicular to the obverse and reverse surfaces of the coin). As noted above, sometimes texture may be found even in an unstruck flan. Similarly, the struck Judaean coins mostly exhibit IFPs with a maximum texture at the [011] orientation expected for compression from hammer strikes (*χ*^2^ = 7.34, *n* = 13, and *P*=0.023).

Figure 12 includes an unexpected IFP for HG-5. This texture and IFP is possibly the result of Pb globules that segregated in the coin during pouring due to gravity [16], though it may also be due to a recrystallization texture caused by annealing. Narrow peak widths in HG-5's spectra (not shown) support this. Thus, it is useful to include multiple lines of evidence when determining thermomechanical histories. For example, it is worth noting that the IFP for JW-4 in Figure 12 indicates this coin was struck as well as annealed, a finding in keeping with the phase width (Figure 10). The metallography (Figure 7(a)) also suggests it was struck while partial annealing was happening [38]. The most likely scenario was that this coin was initially struck when its flan had cooled too far. A poor strike resulted so the minter reheated it to around 200°C to soften it and struck it again. This would also explain the presence of tenorite.

Another surprising IFP (not shown) was obtained from the US-1 flan. It exhibited a pattern in keeping with the struck coins with a small but measurable texture coefficient in the [011] direction. This flan was particularly small, and we subsequently discovered that we had placed it so the hammer or chisel-cut sprue was also in the neutron beam.

The coins in this study are small enough (ca. 1 cm in diameter) that it is possible to strike images at room temperature without heating the cast flans. Because the flans did not need to be flattened, striking, heating (annealing), and then striking these coins again is an unlikely scenario for more than an occasional coin. A single (or even double) strike while the metal is still relatively hot is more probable. The very narrow ND peak widths for AJ-5, JW-3, and JW-4 (spectra not shown) are comparable to the one shown for JW-4, suggesting these three coins may also have been struck while still hot enough to undergo partial annealing during striking of the image. As noted above, HG-5 also exhibits some signs of being struck while hot.

The AJ-3 coin in Figure 7(b) shows slight bending in the microstrain lines that is most consistent with cold striking of the metal. The similar widths and distributions of the *α*-phases for most of the other AJ coins and most of the VG coins suggest they also may have been struck at or below their annealing temperature. With relatively little Pb, these coins' annealing temperatures will be higher than for the coins with significant levels of Pb, so many of these were probably in the “warm” temperature category, having cooled enough they could no longer be considered “hot” strikes. Thus, the presence of little or no Δ texture (Figure 11), annealing texture in the IPFs, and/or narrow diffraction peak widths are all nondestructive indicators of “hot strikes” done near or above the bronzes' annealing temperatures [66]. The combination may be useful as a replacement for the destructive metallographic analyses used heretofore.

The results of this study are consistent with cast flans that were struck sequentially in strips while they were still cooling. While striking coins from hot metal will degrade dies [53], they can be preserved by using more than one die and rotating them through a cooling bath while continuously striking batches of coins. This is in contrast to some Late Roman Empire bronze coins analyzed by Canovaro et al. [15]. The coins they analyzed were made using individual cast flans that were hammered flat and annealed before the images were struck. While the Roman authority coins in this study exhibit major differences in their elemental composition from the Judaean ruler coins, the subsequent differences in the textures and metallography seem to be a result of the lack of Pb in the coin type tested rather than due to major differences in minting techniques. However, the differences in the metal composition of these Roman ruler coins may indicate a different supply chain for the metals used, something which is supported by recent analyses of the isotopic composition of a suite of these Judaean bronze coins [67].

We also find the enhanced levels of surficial Pb compared to the bulk composition, the segregation of Pb, and Pb's impact on the texture analyses and phase widths is consistent with other researchers' findings based on XRD, SEM, metallography [16, 68], and neutron imaging techniques [69].

## 5. Conclusions

TOF-ND provides a more complete multicomponent analysis than is possible with analytical techniques such as XRF, XRD, or microscopy. Its compositional analyses are not compromised by minor surface inhomogeneity created during hammering, wearing, or subsequent corrosion effects, though it also quantifies these components. More importantly, it provides these analyses without destroying the artifact, providing an analysis that includes the interior of the coin. This is important if corrosion products such as nantokite are present but hidden. This mineral is involved in “bronze disease” and it will ultimately destroy an artifact if not mitigated. Detecting and identifying these corrosion products and obtaining a thorough analysis of the bulk composition are very important when developing conservation protocols.

TOF-ND provides insights into methods of manufacture that complement or replace information previously only obtained using destructive analyses. Active research is still being done on interpreting and relating the information obtained by ND to traditional analytical methods. With ongoing improvements in computational methods, neutron flux, and the detectors used in instruments such as VULCAN, future researchers may find applications for its 250 *μ*m spatial resolution of diffraction patterns in small archaeological objects [70]. With dynamic studies at elevated temperatures, it may also be possible in the future to determine the temperature at which ancient metals were struck without the ambiguities encountered in this study.

Finally, by analyzing multiples of four different examples of the same type of coin plus a set of unstruck flans, a better understanding of the population variation and its uncertainty was obtained for the composition, temperature of striking, and method of minting. The differences in the compositions were found to be statistically significant. Because of these differences, striking temperatures may have been varied in these bronzes by the minters to compensate for differences in the hardness of the flans, thus achieving more uniform striking conditions.

## Figures and Tables

**Table 1:** TOF-ND fractions of each phase and their line widths.

COIN ID	Phases (%)				% Sn	Widths (*σ*% Sn)		Texture
	*α*_1_	*α*_2_	Pb	Other	In *α*_2_	*α*_1_	*α*_2_	(MRD)
US1	40.8	50.9	8.4		3.6	0.15	0.12	0.8–1.2
US2	45.6	49.1	4.7		3.5	0.16	0.60	None
US3	35.0	53.1	7.6	3.2^*∗∗*^	7.0	0.27	0.24	None
US4	59.5	40.5	5.7		4.4	0.40	0.60	0.3–2.5
US5	36.1	57.5	5.8		3.6	0.23	0.47	Weak
JH3	61.1	38.0	0.0		2.0	0.16	0.42	0.8–1.3
AJ1	56.0	35.2	1.3	7.6^*∗*^c,n	3.0	0.23	0.60	None
AJ2	65.3	33.2	0.0		1.6	0.21	0.52	0.9–1.1
AJ3	44.0	54.4	0.7		3.7	0.20	0.57	0.4–1.5
AJ4	50.3	46.1	0.0	3.6^*∗*^n	3.7	0.21	0.61	0.6–1.5
AJ5	96.8	<3	0.7	2.5^*∗*^n	<0.2	0.09	<0.05	0.3–1.6
HG1	87.2	<3	9.8	1.5^*∗∗*^	<0.2	0.50	<0.05	None
HG2	29.5	50.6	8.6	10.0^*∗∗∗*^	7.0	0.33	0.34	None
HG3	85.0	<3	4.6	9.6^*∗∗*^	<0.2	0.47	<0.05	0.3–1.5
HG4	90.2	<3	9.7		<0.2	0.57	<0.05	0.8–1.1
HG5	9.2	75.6	2.4	10.7^*∗∗*^	5.9	0.20	0.59	0.4–1.5
VG1	28.1	71.8	0.0		4.7	0.24	0.73	Weak
VG2	29.2	69.5	0.0		4.4	0.27	0.75	0.8–1.2
VG3	48.5	51.5	0.3		6.3	0.24	0.27	0.2–2.0
VG4	28.5	69.8	0.0		4.4	0.20	0.62	0.1–2.6
VG5	28.3	69.6	0.0		4.6	0.23	0.72	0.4–1.5
HA1	65.0	33.2	1.2		3.8	0.17	0.47	N.A.
HA2	31.1	66.8	1.4		4.9	0.29	0.77	0.8–1.6
JW1	34.6	59.8	5.5		6.4	0.25	0.40	0.8–1.2
JW2	36.7	58.3	5.1		3.8	0.28	0.62	0.3–1.5
JW3	76.7	17.8	5.9		2.6	0.41	0.18	None
JW4	13.6	75.5	1.5	9.5^*∗*^c,t	4.1	0.21	0.16	0.4–1.6
JW5	33.1	59.6	6.2		5.5	0.20	0.43	None

Software 2s fitting errors in the phases = 2% and 0.05% Sn in the line widths. ^*∗*^Mineral phases >2% are shown. These include cuprite (c), nantokite (n), and tenorite (t). ^*∗∗*^Cu phase. ^*∗∗∗*^*α*_3_-Bronze. N.A. indicates the sample was not analyzed for its texture.

**Table 2:** TOF-ND (bulk) and XRF (surface) element percentages.

COIN ID	Coin date	ND-Cu	ND-Sn	ND-Pb	XRF-Cu	XRF-Sn	XRF-Pb
US1	No date	87.1	4.51	8.36	77.3	11.3	10.7
US2	No date	93.1	2.23	4.72	83.0	13.0	3.4
US3	No date	86.7	5.66	7.64	88.1	4.3	6.8
US4^*∗*^	No date	90.9	3.45	5.70	83.6	7.1	8.2
US5	No date	91.4	2.75	5.82	88.8	5.5	5.0
JH3^*∗*^	135–104 BCE	98.9	1.10	<0.2	91.8	3.2	4.6
AJ1	80/79 CE	97.1	1.57	1.31	92.0	5.7	1.6
AJ2	80/79 CE	99.2	0.75	<0.2	94.6	2.6	1.6
AJ3^*∗*^	80/79 CE	96.8	2.51	0.65	91.6	6.2	1.3
AJ4	80/79 CE	97.8	2.24	<0.2	92.0	6.2	0.8
AJ5	80/79 CE	96.9	2.44	0.70	92.9	5.1	1.3
HG1	40–4 BCE	84.5	5.64	9.84	80.1	12.0	7.3
HG2^*∗*^	40–4 BCE	86.1	5.36	8.58	80.2	12.8	6.3
HG3	40–4 BCE	90.0	5.39	4.56	73.8	13.8	11.8
HG4	40–4 BCE	85.8	4.55	9.65	76.7	12.3	10.3
HG5	40–4 BCE	92.8	4.82	2.42	71.3	11.0	17.1
VG1	18/19 CE	96.2	3.80	<0.2	85.1	11.5	2.6
VG2^*∗*^	18/19 CE	96.4	3.59	<0.2	89.2	9.6	0.6
VG3	18/19 CE	94.9	4.71	0.35	87.2	11.5	0.5
VG4	18/19 CE	96.3	3.69	<0.2	88.2	10.5	0.5
VG5	18/19 CE	96.3	3.68	<0.2	90.6	8.0	0.8
HA1	41/2 CE	96.8	1.97	1.22	N.A.	N.A.	N.A.
HA2	41/2 CE	94.8	3.81	1.39	N.A.	N.A.	N.A.
JW1^*∗*^	67/68 CE	89.5	4.91	5.55	87.2	6.7	5.3
JW2	67/68 CE	91.9	2.93	5.13	90.1	5.4	3.9
JW3	67/68 CE	90.7	3.39	5.87	84.4	8.4	6.3
JW4^*∗*^	67/68 CE	95.0	3.47	1.50	88.7	5.0	5.6
JW5	67/68 CE	89.8	4.08	6.17	80.0	9.3	9.6

Average instrumental and sampling 2s errors equal 2.8, 0.12, 1.0, 4.5, 1.5, and 3.5 for ND-Cu, ND-Sn, ND-Pb, XRF-Cu, XRF-Sn, and XRF-Pb, respectively. ^*∗*^Coins that were polished on a surface and/or edge. N.A. indicates the sample was not analyzed by XRF.

## Data Availability

The data used to develop the findings of this study are summarized in Tables 1 and 2. The original ND files are archived at ORNL, specifically, dataset numbers 9422–9434 and 9971–10635.
